# Methicillin-Resistant Staphylococcus aureus Bacteremia Originating From a Ureteral Stent-Associated Urinary Tract Infection: A Case Report

**DOI:** 10.7759/cureus.113469

**Published:** 2026-07-27

**Authors:** Yoshihiro Kawaguchi, Junichi Kurogi

**Affiliations:** 1 Department of Urology, Saiseikai Futsukaichi Hospital, Chikushino, JPN; 2 Department of Urology, Morodomi Central Hospital, Saga, JPN; 3 Department of Gastroenterology, Saiseikai Futsukaichi Hospital, Chikushino, JPN

**Keywords:** bacteremia, methicillin-resistant staphylococcus aureus, staphylococcus aureus, ureteral stent, urinary tract infection

## Abstract

Methicillin-resistant *Staphylococcus aureus* (MRSA) is an uncommon cause of urinary tract infection but can result in severe systemic complications, particularly in patients with indwelling urinary devices. We report a case of MRSA bacteremia associated with a ureteral stent in an 83-year-old woman who presented with a high-grade fever. Computed tomography demonstrated left hydronephrosis despite stent placement. Emergency stent exchange revealed whitish purulent debris in the bladder and draining from the stent side holes. Blood, bladder urine, and renal pelvic urine cultures all yielded MRSA. Targeted therapy with teicoplanin led to rapid clinical improvement and microbiological clearance. This case highlights that ureteral stents may serve as a source of MRSA bacteremia even in the absence of complete obstruction, emphasizing the importance of prompt source control and appropriate antimicrobial therapy.

## Introduction

Methicillin-resistant *Staphylococcus aureus* (MRSA) bacteremia is associated with substantial morbidity and mortality and requires prompt identification of the infectious source and appropriate antimicrobial therapy [1,2]. Although MRSA is a well-recognized pathogen in bloodstream infections, it is an uncommon cause of urinary tract infections, accounting for only a small proportion of cases [3,4]. MRSA accounts for approximately 0.5%-6% of urinary tract infections, although the reported prevalence varies among patient populations and healthcare settings [3,4]. MRSA urinary tract infections are more frequently observed in elderly patients and those with indwelling urinary devices, suggesting the important role of device-associated colonization and biofilm formation [3-5].

A ureteral stent is a thin tube placed between the kidney and bladder to maintain urinary drainage in patients with ureteral obstruction. Although indispensable in modern urological practice, ureteral stents serve as foreign bodies to which microorganisms readily adhere, promoting biofilm formation and making eradication of infection more difficult [5,6].

Ureteral stents are widely used in urological practice but may serve as a nidus for microbial colonization and persistent infection because biofilms can develop on their external surfaces and within their lumens [5,6]. However, reports specifically describing MRSA bacteremia originating from ureteral stent-associated urinary tract infection remain extremely limited. We report a case of MRSA bacteremia originating from a ureteral stent-associated urinary tract infection and discuss its clinical implications.

## Case presentation

An 83-year-old woman with a history of dementia, chronic kidney disease, and chronic hepatitis B treated with tenofovir alafenamide was transported to our hospital because of a high-grade fever. Because of advanced dementia, detailed urinary symptoms, including dysuria, urinary frequency, hematuria, and flank pain, could not be reliably assessed. A ureteral stent had been placed five months earlier for left calculous pyelonephritis, and the first stent exchange had been performed 86 days before admission. She was due for a scheduled stent exchange at the time of presentation. On admission, her vital signs and laboratory findings are summarized in Table 1.

**Table 1:** Vital signs and laboratory findings on admission. *Reference range for adult women.

Parameter	Result	Reference range
Vital signs		
Body temperature	40.2 ℃	36.0-37.5 ℃
Blood pressure	134/64 mmHg	ー
Heart rate	120 beats/min	60-100 beats/min
Laboratory findings		
White blood cell count	18000/µL	3300-8600/µL
C-reactive protein	7.84 mg/dL	0-0.14 mg/dL
Serum creatinine	2.00 mg/dL	0.46-0.79 mg/dL*
Venous lactate	2.4 mmol/L	0.5-2.0 mmol/L

**Figure 1:**
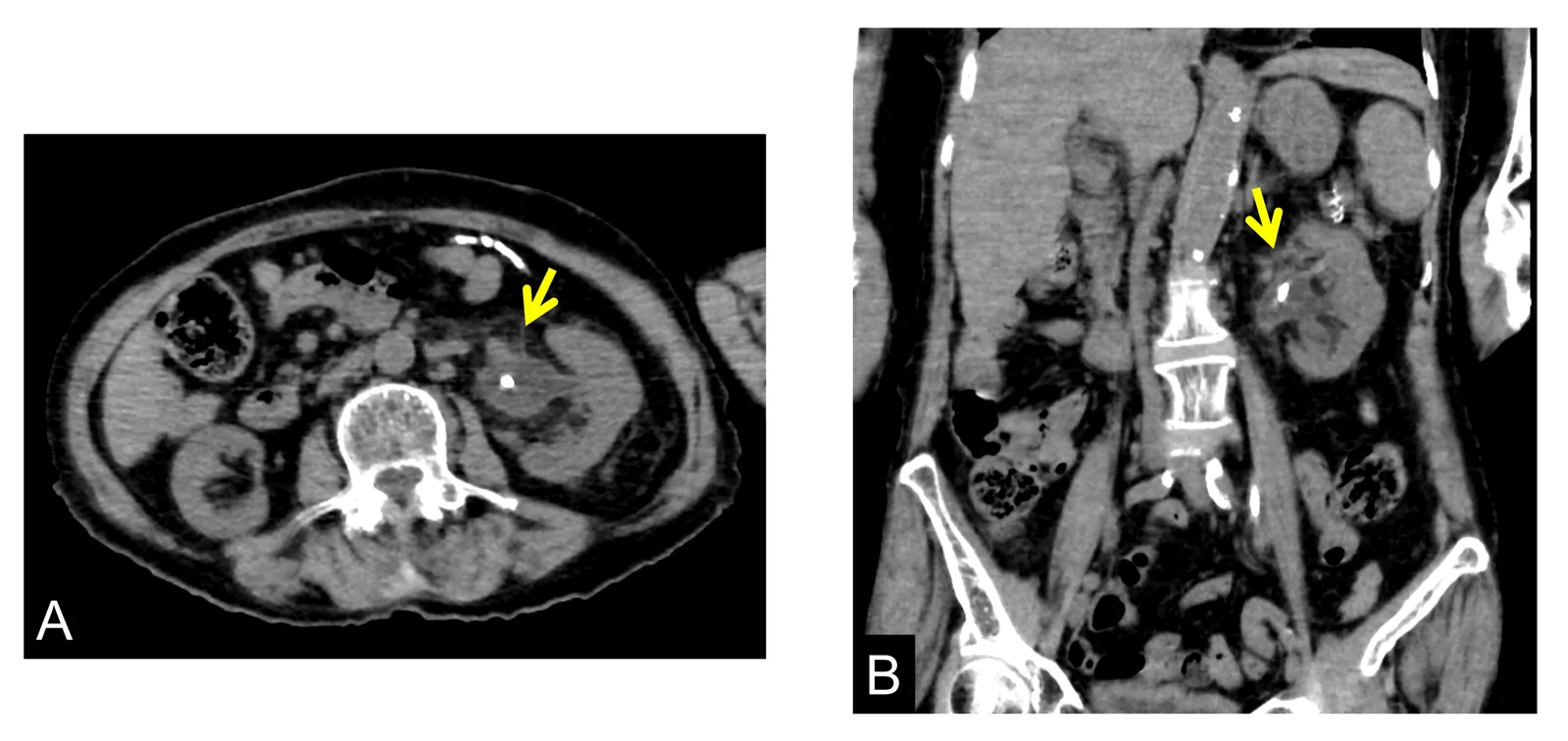
Computed tomography demonstrating left hydronephrosis despite an indwelling ureteral stent. A) Axial computed tomography image showing left hydronephrosis despite the presence of a ureteral stent. The yellow arrow indicates the dilated left renal pelvis. B) Coronal computed tomography image demonstrating left hydronephrosis and the indwelling ureteral stent. The yellow arrow indicates left hydronephrosis.

Computed tomography demonstrated left hydronephrosis and increased perirenal fat stranding around the left kidney despite the presence of a ureteral stent, suggesting stent obstruction (Figure 1).

**Figure 2:**
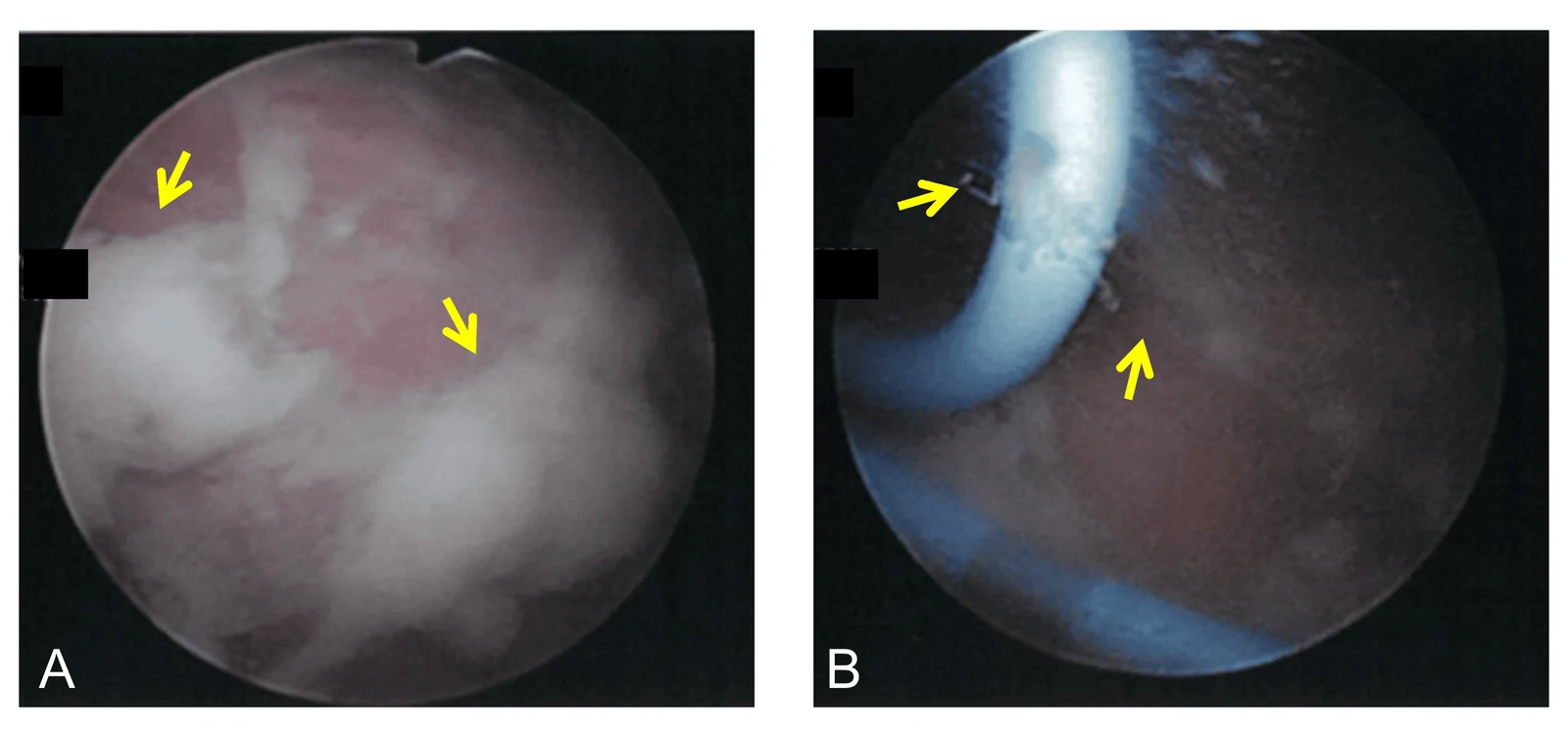
Intraoperative findings during ureteral stent exchange. A) Whitish debris observed in the bladder during cystoscopy. The yellow arrows indicate whitish purulent debris within the bladder. B) Whitish purulent material draining from the side holes of the ureteral stent. The yellow arrows indicate purulent material draining from the side holes of the ureteral stent.

Emergency ureteral stent exchange was performed on the day of admission. During the procedure, whitish purulent debris was observed in the bladder and draining from the side holes of the ureteral stent (Figure 2).

Approximately 7 mL of turbid urine was collected from the renal pelvis. Cefmetazole was initiated as empirical antimicrobial therapy.

MRSA was isolated from blood, bladder urine, and renal pelvic urine cultures. The antimicrobial susceptibility profile of the MRSA isolate is summarized in Table 2.

**Table 2:** Antimicrobial susceptibility profile of the methicillin-resistant Staphylococcus aureus isolate.

Antimicrobial agent	Susceptibility	Minimum inhibitory concentration (µg/mL)
Vancomycin	Susceptible	1
Teicoplanin	Susceptible	<=4
Linezolid	Susceptible	<=2

**Figure 3:**
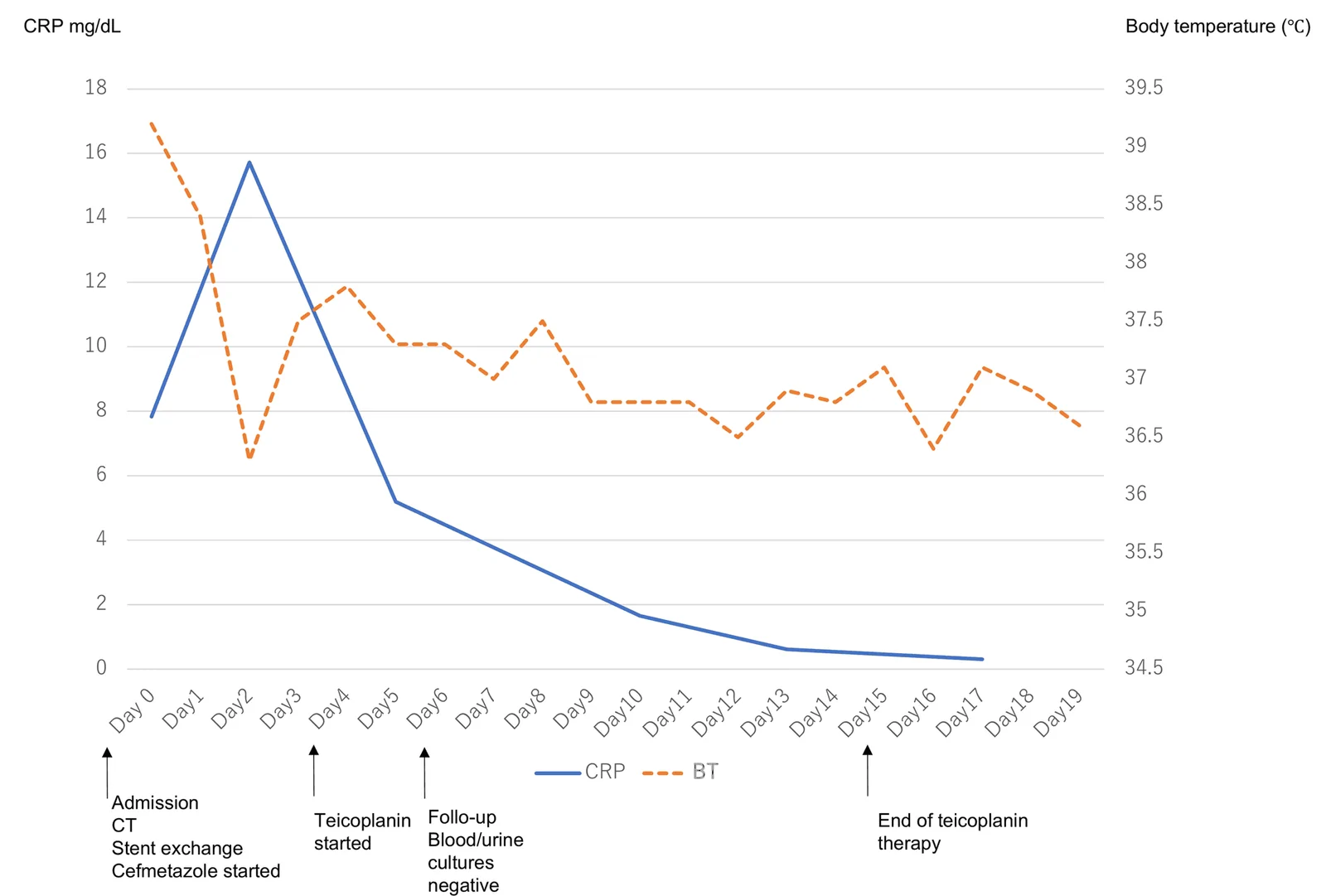
Clinical course and treatment response. Clinical course showing changes in body temperature and C-reactive protein together with key clinical events, including admission, computed tomography, ureteral stent exchange, initiation of antimicrobial therapy, follow-up negative cultures, and completion of treatment. Abbreviations: CRP, C-reactive protein; BT, body temperature.

Based on these results, antimicrobial therapy was changed to teicoplanin on hospital day 4. The trough concentration of teicoplanin was maintained within the therapeutic range (24.2-24.9 μg/mL). The patient showed rapid clinical improvement, and follow-up blood and urine cultures obtained on hospital day 6 were negative. Transthoracic echocardiography revealed no evidence of infective endocarditis. Because follow-up blood cultures became negative promptly and transthoracic echocardiography showed no evidence of infective endocarditis, transesophageal echocardiography was not performed. Owing to the patient's underlying dementia, a comprehensive assessment for symptoms suggestive of metastatic infection was limited. Teicoplanin was continued for 15 days based on documented clearance of bacteremia, a favorable clinical response, and current management principles for uncomplicated MRSA bacteremia. The near normalization of the C-reactive protein level supported the patient's clinical improvement (Figure 3).

The patient's subsequent clinical course was uneventful, and she was transferred to a rehabilitation hospital on hospital day 31.

## Discussion

This case provides two important clinical insights. First, ureteral stents can serve as a nidus for colonization and subsequent MRSA bacteremia, even in the absence of complete obstruction. Second, prompt source control through stent exchange combined with targeted anti-MRSA therapy is essential for successful management.

MRSA bacteremia is associated with substantial morbidity and mortality, and its management requires early identification of the infectious source, appropriate antimicrobial therapy, and confirmation of microbiological clearance using follow-up blood cultures [1,2]. Source control, including the removal or exchange of infected devices, is a key determinant of favorable clinical outcomes [1].

Although MRSA is a well-recognized cause of bloodstream infections, it accounts for only a small proportion of UTIs [3,4]. MRSA urinary tract infections occur more frequently in elderly patients and in those with indwelling urinary devices, suggesting that device-associated colonization and biofilm formation play important roles in their pathogenesis [3-5].

Previous studies have demonstrated that microbial biofilms can develop not only on the external surfaces but also within the lumens of ureteral stents, potentially serving as a persistent source of infection even in the absence of complete obstruction [5,6]. In the present case, MRSA was consistently isolated from blood, bladder urine, and renal pelvic urine, strongly supporting the urinary tract as the source of bacteremia. Furthermore, whitish purulent debris was directly visualized in the bladder and draining from the side holes of the ureteral stent during exchange. Although these findings are not specific to MRSA, they are compatible with device-associated infection and may reflect underlying biofilm formation.

Rapid clinical improvement and microbiological clearance were achieved following stent exchange and targeted teicoplanin therapy. This clinical course is consistent with current principles of MRSA bacteremia management, which emphasize the importance of effective antimicrobial treatment and timely source control [1,2]. According to current guidelines, the duration of antimicrobial therapy for uncomplicated *Staphylococcus aureus* bacteremia should be determined based on documented clearance of bacteremia and clinical response rather than normalization of inflammatory markers such as C-reactive protein [1,2].

To our knowledge, reports clearly documenting MRSA bacteremia originating from ureteral stent-associated urinary tract infection remain scarce. The present case is notable because MRSA was simultaneously isolated from blood, bladder urine, and renal pelvic urine, and purulent debris suggestive of device-associated infection was directly observed during ureteral stent exchange. These findings provided compelling evidence supporting the urinary tract as the primary source of bacteremia and further emphasize the importance of considering ureteral stents as a potential source of MRSA bacteremia.

Following prompt stent exchange and targeted teicoplanin therapy, rapid clinical improvement and microbiological clearance were achieved. This clinical course underscores the importance of combining effective antimicrobial treatment with timely source control, particularly optimizing therapeutic drug monitoring for teicoplanin to ensure favorable outcomes in MRSA bacteremia [7]. Although routine empirical anti-MRSA therapy is not recommended for all patients with urinary tract infections, it may be considered in selected high-risk patients, particularly elderly individuals with indwelling urinary devices presenting with severe sepsis or septic shock, until microbiological results become available.

This report has some limitations. First, this was a single-case report, and the findings may not be generalizable to other patients. Second, although the clinical course strongly suggested that the ureteral stent was the source of infection, direct microbiological confirmation of biofilm formation on the stent was not obtained.

## Conclusions

This case highlights the importance of considering ureteral stents as a potential source of MRSA bacteremia even when complete obstruction is not evident. Prompt source control and targeted antimicrobial therapy are essential for successful management. Clinicians should consider indwelling ureteral stents as a potential source of MRSA bacteremia in patients presenting with severe urinary tract infections.
